# A guide to *Agrobacterium*-mediated transformation of the chytrid fungus *Spizellomyces punctatus*

**DOI:** 10.1099/acmi.0.000566.v3

**Published:** 2023-05-11

**Authors:** Sarah M. Prostak, Edgar M. Medina, Erik Kalinka, Lillian K. Fritz-Laylin

**Affiliations:** ^1^​ Department of Biology, University of Massachusetts Amherst, Amherst, MA, USA

**Keywords:** Agrobacterium-mediated transformation, *Agrobacterium*, *Spizellomyces punctatus*, chytrid fungi, chytrids, transformation, genetic tools

## Abstract

Chytrid fungi play key ecological roles in aquatic ecosystems, and some species cause a devastating skin disease in frogs and salamanders. Additionally, chytrids occupy a unique phylogenetic position– sister to the well-studied Dikarya (the group including yeasts, sac fungi, and mushrooms) and related to animals– making chytrids useful for answering important evolutionary questions. Despite their importance, little is known about the basic cell biology of chytrids. A major barrier to understanding chytrid biology has been a lack of genetic tools with which to test molecular hypotheses. Medina and colleagues recently developed a protocol for *Agrobacterium *-mediated transformation of *Spizellomyces punctatus*. In this manuscript, we describe the general procedure including planning steps and expected results. We also provide in-depth, step-by-step protocols and video guides for performing the entirety of this transformation procedure on protocols.io (dx.doi.org/10.17504/protocols.io.x54v9dd1pg3e/v1).

## Data Summary

The authors confirm all supporting data, code and protocols have been provided within the article or through supplementary files. Supplementary materials include detailed steps for the entire protocol discussed in this manuscript. These steps can also be found on protocols.io (dx.doi.org/10.17504/protocols.io.x54v9dd1pg3e/v1).

## Introduction

Chytrids are early-branching, largely aquatic fungi of great ecological importance [1]. While many chytrid species are free-living saprobes [2], others are deadly parasites of diatoms, algae, and some vertebrates [3]. One species, *Batrachochytrium dendrobatidis* (*Bd*), has garnered widespread attention as the ‘frog-killing fungus’, causing a deadly skin infection that is devastating frog populations around the world [4, 5]. Moreover, their phylogenetic position places them towards the base of the fungal lineage, having diverged before the diversification of the Dikarya (the group including yeasts, sac fungi, and mushrooms). This phylogenetic position, along with the retention of both fungal and ancestral traits like motile cilia (also called flagella) [6], make them especially useful for answering key evolutionary questions [1].

Despite their clear importance, little is known about the molecular and cell biology of chytrids. The major barrier for answering mechanistic questions in chytrids is a lack of genetic tools to use in the lineage. Recently, Medina and colleagues developed the first and only protocol to stably express transgenes in the chytrid species *Spizellomyces punctatus* (*S.p*.) using *Agrobacterium tumefaciens-*mediated transformation [7]. This technique opens the door to investigating chytrid pathogenesis and broadens researchers’ abilities to explore the evolution of animals, fungi, and their unicellular relatives.

Chytrids have a biphasic life cycle, spending the first part of their life as a motile zoospore with a singular posterior flagellum and no cell wall. Motile zoospores eventually settle, retract their flagellum, build a cell wall, and grow to become sessile, reproductive sporangia. Sporangia undergo multiple rounds of mitosis without cytokinesis before dividing their cytoplasm to produce the next generation of mononuclear zoospores that are released back into the environment. *S.p*. undergoes a complete life cycle from single zoospore to release of 20–50 zoospores in just 18–20 h when grown at 28 °C on K1 media (w/v: 0.06 % peptone; 0.04 % yeast extract; 0.18 % glucose; 1.5 % agar). These simple growth conditions make *S.p*. an excellent organism for use in the laboratory.

*S.p*. transformation relies on *Agrobacterium tumefaciens (A.t*.), a plant pathogen that has been co-opted to genetically modify a wide variety of organisms. Infection by *A.t*. results in transfer of *A.t*. DNA into the plant genome that induces tumours and changes plant metabolism [8]. This organism and its infection strategy has been adapted for genetic manipulation of many plants and fungi [9, 10]. *S.p*. is currently the only chytrid that can be genetically modified by *Agrobacterium *-mediated transformation [7].

Here, we provide an overview of the protocol for *A.t*.-mediated transformation of *S.p*. A detailed collection of experiment steps, materials, timing, and precautions is available at protocols.io (dx.doi.org/10.17504/protocols.io.x54v9dd1pg3e/v1). A roughly 40 min video of the entire protocol is also associated with the entry on protocols.io to help demonstrate the entire process, focusing on the more intricate steps. We hope that this document and its accompanying materials will aid in the dissemination of knowledge at this pivotal point in the history of chytrid research and the broader field of evolutionary research.

## Advanced planning

Generally, *S.p*. transformation includes the following steps: 1) transforming *A.t*. with a plasmid of interest; 2) growing transformed *A.t*. to an OD660 of 0.6; 3) co-culturing *S.p*. zoospores and *A.t*. on a medium that induces the bacteria’s virulence genes; 4) selecting for transformed *S.p*. using antifungal compounds; and 5) picking transformed colonies and culturing them.

**Fig. 1.**
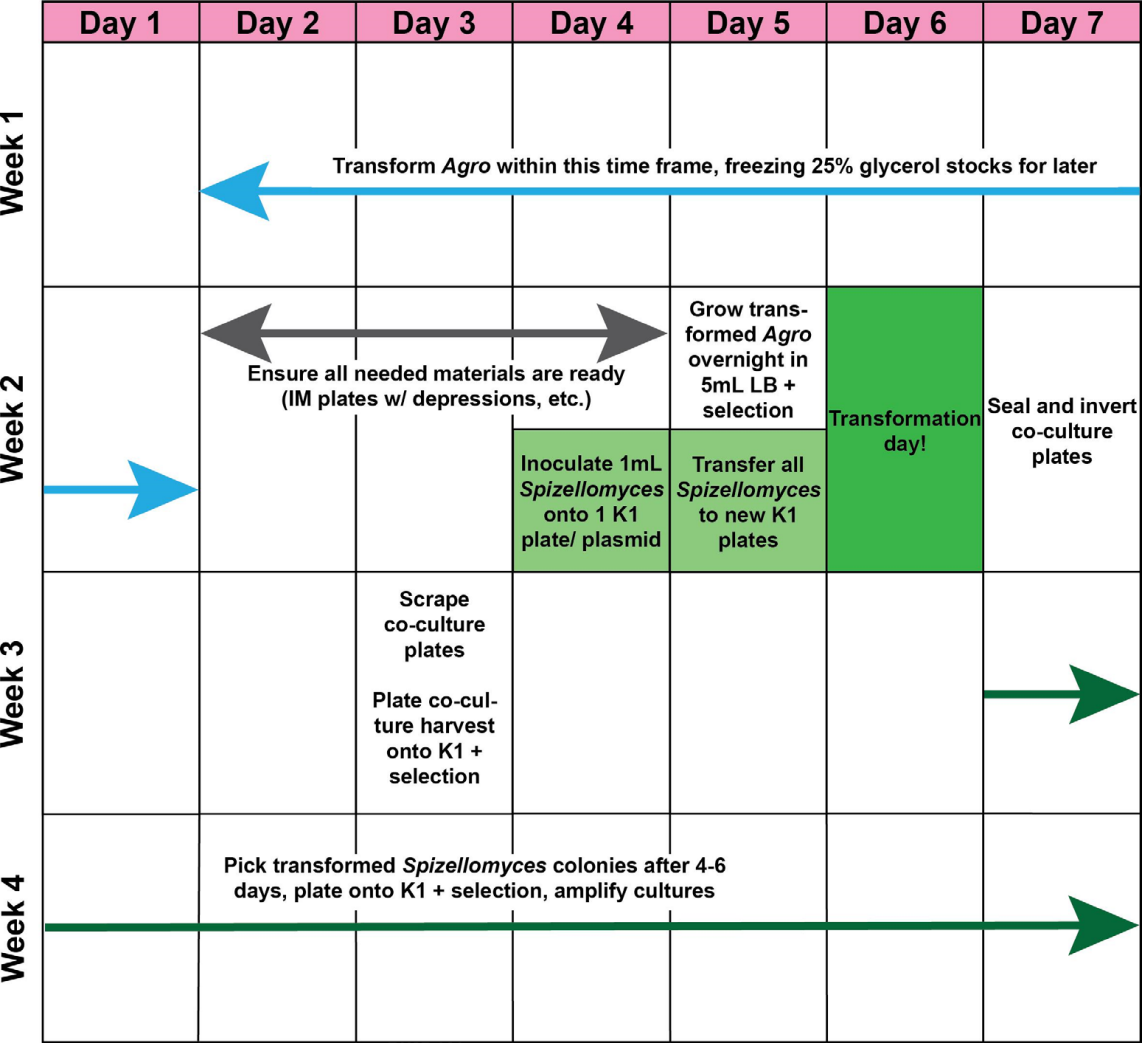
Overview of *Agrobacterium *-mediated *Spizellomyces punctatus* transformation. A general timeline of the different steps involved in the transformation of *Spizellomyces punctatus* (*S.p*.) by *Agrobacterium tumefaciens (Agro, A.t.). A.t*. should generally be electroporated with the plasmid of interest at least 4 days prior to the planned transformation time to ensure that pickable colonies are obtained. All materials should be prepared 3–4 days prior to transformation day. *S.p*. should be subcultured onto antimicrobial-free K1 media 36 and 18 h prior to the intended transformation time. Following transformation day, seal and invert co-culture plates. About 4 days after transformation day, harvest the co-culture plates and select transformants by plating on selective K1 media. Cells will need to grow for 4–6 days after being plated onto selection media before colonies appear (if the transformation was successful). Overall, the process takes about 4 weeks from electroporating *A.t*. to having a stable culture of transformed *S.p*.

This procedure involves many steps that span nearly a month and require significant advance planning. Fig. 1 outlines an efficient timeline for the entire procedure, including advance preparation of necessary materials. Of particular importance is allowing sufficient time for *A.t*. growth; *A.t*. lawns, colonies, or liquid cultures should not be used for any step of this procedure unless they have been growing at 28 °C for at least 48 h. These conditions are based on our laboratory set up, and further optimization from the community is welcomed. The number of each type of plate required per plasmid to be transformed is outlined in Table 1.

**Table 1.** The number and types of agar plates required per plasmid for *Agrobacterium *-mediated transformation of *Spizellomyces punctatus*

	no. of plates per plasmid				
Protocol Step	LB	LB+selection	K1	K1+selection	IM w/ depressions
A.t. Electroporation	1	1	–	–	–
Culturing S.p. 36 h before transformation	–	–	1	–	–
Culturing S.p. 18 h before transformation	–	–	1	–	–
Co-culturing A.t. and S.p.	–	–	–	–	1
Selecting for S.p.transformants	–	–	1	1	–
Picking colonies of transformants	–	–	–	one per four colonies	–
Total # of plates per plasmid	1	1	3	2	1

A.t., Agrobacterium tumefaciens; S.p., Spizellomyces punctatus.

Several growth times must be considered when planning steps before and on transformation day. Prior to transformation, *S.p*. cultures must be semi-synchronized and grown on antimicrobial-free media for at least two generations. To synchronize the population, *S.p*. should be subcultured roughly 36 and 18 h prior to your planned transformation time. On transformation day, *A.t*. needs to grow to an OD660 of 0.6, which in our experience takes about 4 h. The timing for growth to the proper OD should be empirically tested for each laboratory. Additionally, harvesting *S.p*. zoospores on transformation day takes about 1 h and should be coordinated so that *A.t*. and *S.p*. zoospores are ready around the same time.

After transformation day, you will need to return 12–24 h later to seal and invert the co-culture plates. It will take roughly 4 days to see any growth on these co-culture plates. Selecting for transformants takes another 4–6 days before the appearance of *S.p*. colonies on selection media. Once colonies appear, amplification of the colonies to grow enough cells for downstream applications can take up to another week. Overall, it will take about 4 weeks to go from wild-type *A.t*. to stably transformed *S.p*. cultures. In our experience, this method is successful over 80 % of the time.

## Methods

The protocols described in this article are published in detail on protocols.io (dx.doi.org/10.17504/protocols.io.x54v9dd1pg3e/v1) and are included in the supplement here for printing (File. S1, available in the online version of this article). A roughly 40 min video detailing the entire *S.p*. transformation protocol–from electroporation of *A.t*. to selecting for and culturing *S.p*. transformants–can also be found on protocols.io.

### Growing liquid cultures prior to transformation day

We prepared competent *Agrobacterium tumefaciens* EHA105 (*A.t*.; GoldBio #CC-225–5×50) cells at 4 °C with cold reagents, as described in Weigel and Glazebrook [11]. Briefly, we grew *A.t*. at 28°C for at least 48 h before harvesting the cells and washing three times with water. We resuspended cells into sterile 10 % glycerol and transformed them with a plasmid of interest using a 2 mm cuvette and a GenePulser exponential decay electroporator (BioRad, USA) with the following settings: 25 µF, 200 Ω, 2.5 kV. We have had success with plasmids derived from the plasmid pPZP201-BK [12]. Cells recovered in SOC medium by shaking at 225 r.p.m. for 4 h at 28°C. We then plated the cells onto LB plates with selection antibiotics (we typically use 50 mg l^−1^ kanamycin) and grew at 28°C for 4 days or until individual colonies appeared. We picked colonies into 5 ml LB broth each with selection antibiotics and grew the cultures overnight at 28°C and shaking at 225 r.p.m. We recommend freezing 0.5 ml of this culture in 25 % glycerol at −80°C. In case the *Spizellomyces* transformation is unsuccessful, you can streak from this stock for colonies of the transformed *A.t*. The rest of the culture we used in co-culturing of *A.t*. and *S.p*., described later.

### Culturing *Spizellomyces punctatus* prior to transformation day

Unless otherwise noted, we grew *Spizellomyces punctatus* Koch type isolate NG-3 Barr (*S.p*.; ATCC 48900) cultures at room temperature on K1 agar plates [0.06 % bacto peptone (w/v; BD #211677), 0.04 % yeast extract (w/v; Fisher #BP1422-2), 0.18 % glucose (w/v; Sigma #G5767-5KG), 1.5 % agar (w/v; Fisher #BP1423-500)]. About 36 and 18 h before the intended *S.p*. transformation time, we subcultured *S.p*. onto one fresh K1 plate per plasmid to be transformed.

### *Agrobacterium *-mediated transformation of *Spizellomyces punctatus*

We diluted overnight *A.t*. liquid cultures to an OD660 of 0.15 in induction media [1 x minimal salts [13], 40 mM 2-(N-morpholino)ethanesulfonic acid (MES; Sigma #M2933-500G) pH 5.3, 10 mM glucose, 0.5 % (v/v) glycerol (Fisher #G33-500), 200 µM acetosyringone (Sigma #D134406-5G)]. We then grew the cells to an OD600 of 0.6 by shaking at 225 r.p.m. for 4 h at 28 °C.

Meanwhile, we harvested *S.p*. zoospores by flooding culture plates with induction media (IM) or Dilute Salts (DS) solution [14] for 1 h before pooling all zoospores together. We then passed the suspension through a 40 µM mesh filter and then again through a sterile syringe filter with Whatman grade one filter paper. We aimed for a filtered zoospore concentration between 1×10^6^ and 1×10^7^ cells ml^−1^, centrifuging the cells at 2000 rcf for 5 min and resuspending into IM if needed.

**Fig. 2.**
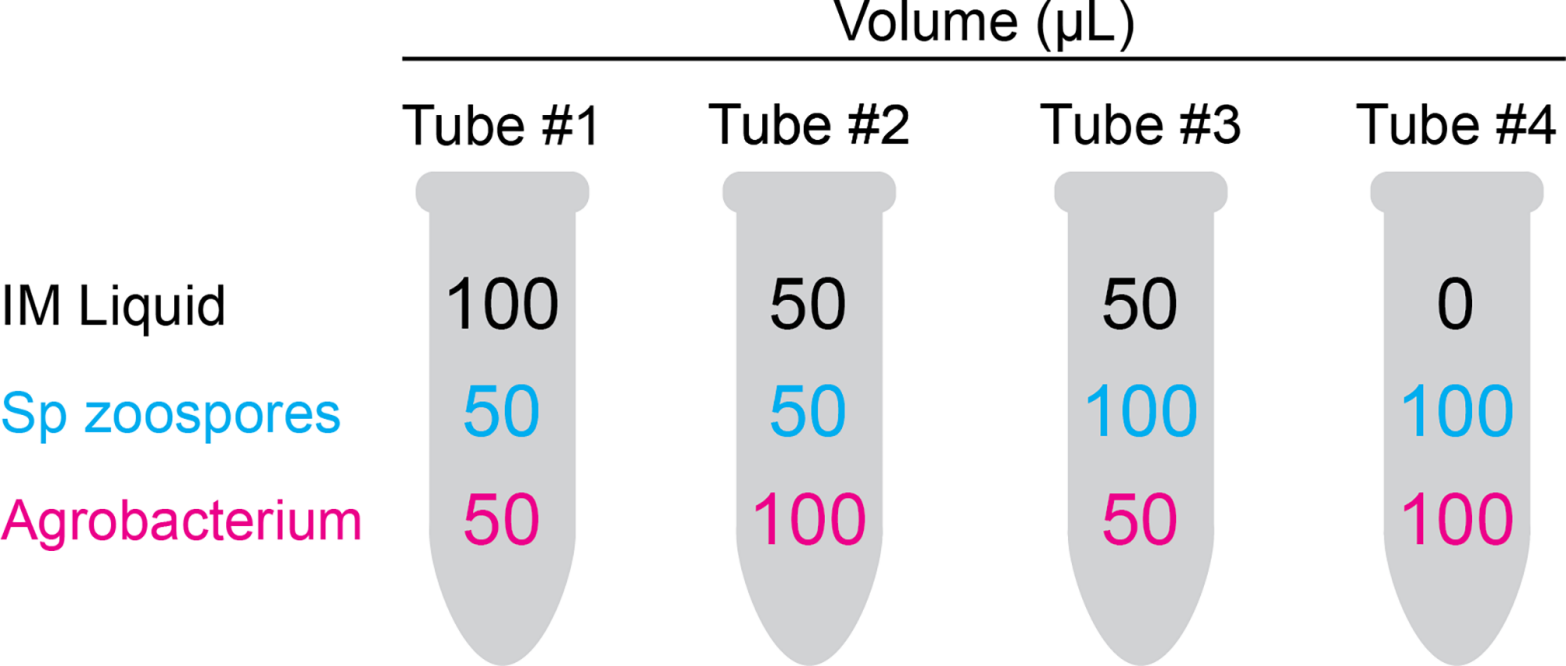
Co-culture ratios of *Agrobacterium tumefaciens* and *Spizellomyces punctatus*. Using several ratios of *Agrobacterium tumefaciens (A.t.) to Spizellomyces punctatus (S.p*.) for co-culturing increases the chances of successful transformation. This is necessary due to the natural variation in zoospore release from day-to-day. Set up four 1.5 ml microcentrifuge tubes per plasmid with the indicated volumes of either IM liquid, *S.p*. zoospores (aim for between 1×10^6^ and 1×10^7^ cells ml^−1^), and *A.t*. at an OD660 of 0.6. To prevent cross contamination of plasmids, fill the tubes in this order: IM then *S.p*. then *A.t*.

For each plasmid to be transformed, we mixed *A.t*. (at OD660 0.6) and *S.p*. zoospores (filtered) in four ratios in IM (Fig. 2), at a final volume of 200 µl. We then plated the total volume of each ratio onto one quadrant of an IM plate. To ensure a tight contact between the *A.t*. and *S.p*. cells, we made a roughly 1-inch diameter depression in each quadrant of an IM plate, created by gently pressing a warm, sterile, round-bottomed glass tube into the surface of the agar. We left the plates unsealed until the co-culture liquid dried (about 12–24 h) and then sealed and incubated them at room temperature for 4 days.

### Selecting for *Spizellomyces punctatus* transformants

After the co-culture plates grew for 4 days, we rehydrated each quadrant with ~250 µl of DS. To harvest the cells, we scraped the surface of the agar with a sterile, single edged razor blade, washed the plate three times with 1 ml of DS, each time adding the liquid to the same 50 ml conical tube. We then brought the volume of the tube to 30 ml with DS. We mixed the cells by inverting the tube three times and vortexed it for 1–2 s to dislodge any *A.t*. still attached to *S.p*. We then pelleted the cells at 2000 rcf for 10 min and gently poured off the supernatant and resuspended the cells into 500 µl of DS. Then, we plated 200 µl of resuspended cells onto K1 plates with selection antimicrobials (300 mg l^−1^ hygromycin B to select for *S.p.* transformants, 50 mg l^−1^ carbenicillin and 50 mg l^−1^ tetracycline to prevent *A.t*. growth). Once the plates were dry, we incubated them in a humidity chamber at room temperature for 4 days until individual colonies appeared. The number of colonies varies; we typically obtain 5–100 *S.p*. colonies per transformation.

### Picking colonies of transformed *Spizellomyces punctatus*

Once colonies of *S.p*. appeared on selection media, we picked and grew a few of them for downstream validation and analysis. We picked colonies by gently lifting them from the agar with a sterile 18G needle and resuspending each into 50 µl of DS. We gently broke up the colony by pipetting and then plated the colony suspension onto selective K1 media. To save time and materials, we plated up to four colonies from the same plasmid transformation onto one quadrant each of a single plate. After 2–3 days we rehydrated each of these quadrants with 100 µl of DS and then transferred them to their own K1 plate. We continued subculturing until there was enough culture to freeze and also to continue with downstream procedures. The method used to confirm success of transformation depends on the downstream applications. We typically use PCR validation of the hygromycin resistance gene, but we also use western, northern, and Southern blotting and/or fluorescence microscopy if the gene of interest has a fluorescent tag [1].

## Expected results

### *Agrobacterium tumefaciens* electroporation & growing liquid cultures prior to transformation day

**Fig. 3.**
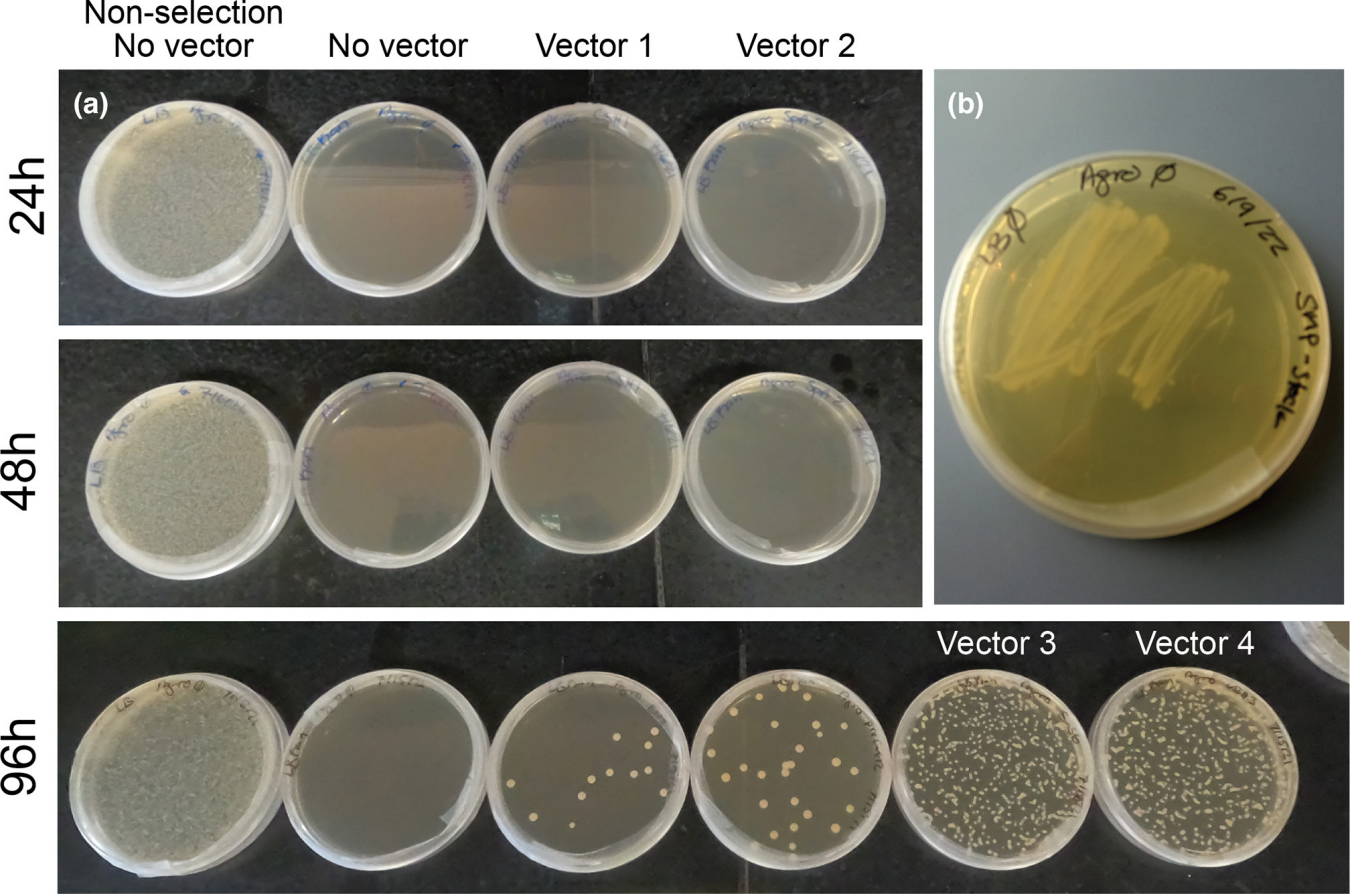
Growth of *Agrobacterium tumefaciens* on LB media. (**a**) *Agrobacterium tumefaciens (A.t*.) was electroporated with no vector or vector 1, 2, 3, or 4. No vector controls were plated on non-selective and selective LB media. Cells electroporated with each vector were plated on selective LB media. Plates were incubated at 28°C and imaged at 24, 48, and 96 h post-plating to show the progression of colony growth. Different transformations yield varying colony morphologies. Visible growth does not typically occur until 72–96 h after plating. (**b**) Streak of wild-type *A.t*. on non-selective media. The overall appearance of growth on the plate is similar to that of streaks of transformed strains of *A.t*. on selective media.

Successful electroporation of *A.t*. is evidenced by the presence of colonies after 72–96 h on selective LB media (Fig. 3a). Colonies can vary in number and size, ranging from 0.1 to 1 mm. Make sure to plate a no vector electroporation sample on both selective and non-selective media controls. This step is to check if the cells survived the electroporation and to check for inherent selection resistance in the competent cells. When creating streaked plates from frozen stock of the desired *A.t*. strain, the plate should look similar to that in Fig. 3b. Liquid cultures of *A.t*. should be cloudy if growth was successful.

### Culturing *Spizellomyces punctatus* prior to transformation day

Determining the health of the *S.p*. culture prior to transformation day is important. An unhealthy culture will not yield as many transformants. *S.p*. cultures are healthy if there are active, swimming zoospores and some free spaces between sporangia when viewed under a microscope.

### *Agrobacterium *-mediated transformation of *Spizellomyces punctatus*

**Fig. 4.**
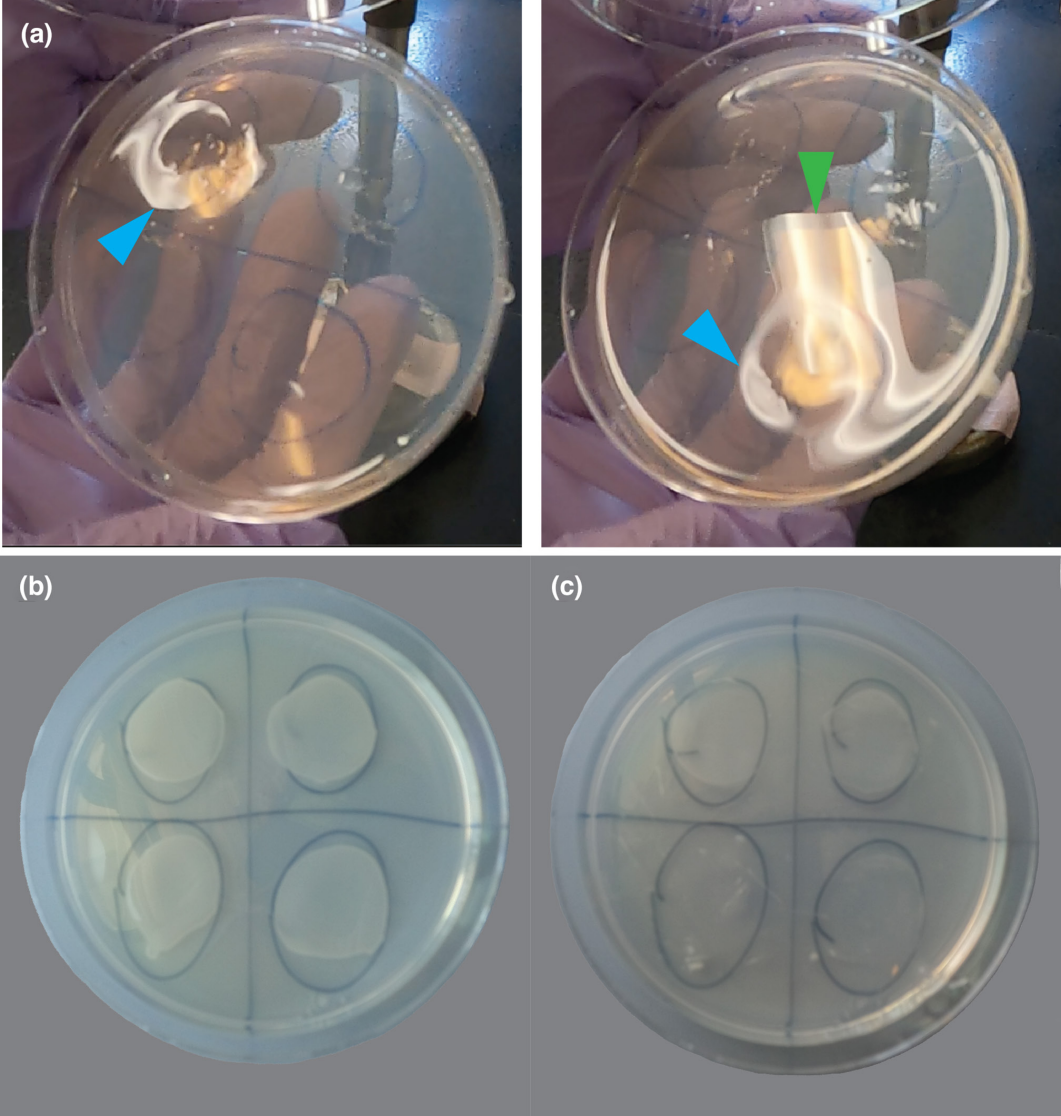
Evaluation of depressions and co-culturing *Agrobacterium tumefaciens* and *Spizellomyces punctatus* on IM plates. (**a**) These ~1 inch in diameter depressions are made to ensure tight contact between *Agrobacterium tumefaciens* and *Spizellomyces punctatus* cells. As light hits depressed agar, it reflects around the curved perimeter (blue arrows), while light that hits non-depressed agar remains fairly linear (green arrow). This is a simple method to evaluate the size of the depressions created in the agar (see Methods and dx.doi.org/10.17504/protocols.io.x54v9dd1pg3e/v1). (**b**) Image of a co-culture IM plate after 4 days of incubation at room temperature. Growth appears as opaque areas on the agar. (**c**) Image of the same plate in (**b**) after harvesting cells by scraping with a sterile razor blade (see Methods and dx.doi.org/10.17504/protocols.io.x54v9dd1pg3e/v1). Nearly all of the opaque areas should be removed to increase chances of recovering transformed *Spizellomyces punctatus*.

Creating depressions that can hold 200 µl of co-culture is important in increasing the chances of transformation success. Depressions in the fairly clear IM plates used for co-culturing *A.t.* and *S.p*. can be difficult to see. Holding the plate at an angle against the light allows for better detection of the depressions (Fig. 4a). Make sure to maintain sterility of the plate while doing this. The light will bend around the perimeter of the depression (Fig. 4a, blue arrows), but will stay nearly linear when hitting non-deformed agar (Fig. 4a, green arrows).

Once the depressions are the right size (about an inch in diameter and several millimetres deep), these IM plates will be used to co-culture *A.t*. and *S.p*. After 4 days at room temperature, the plates will be ready to harvest if opaque growth is present (Fig. 4b). After the harvesting process, nearly all of the growth should be removed from the plate (Fig. 4c).

### Selecting for *Spizellomyces punctatus* transformants

**Fig. 5.**
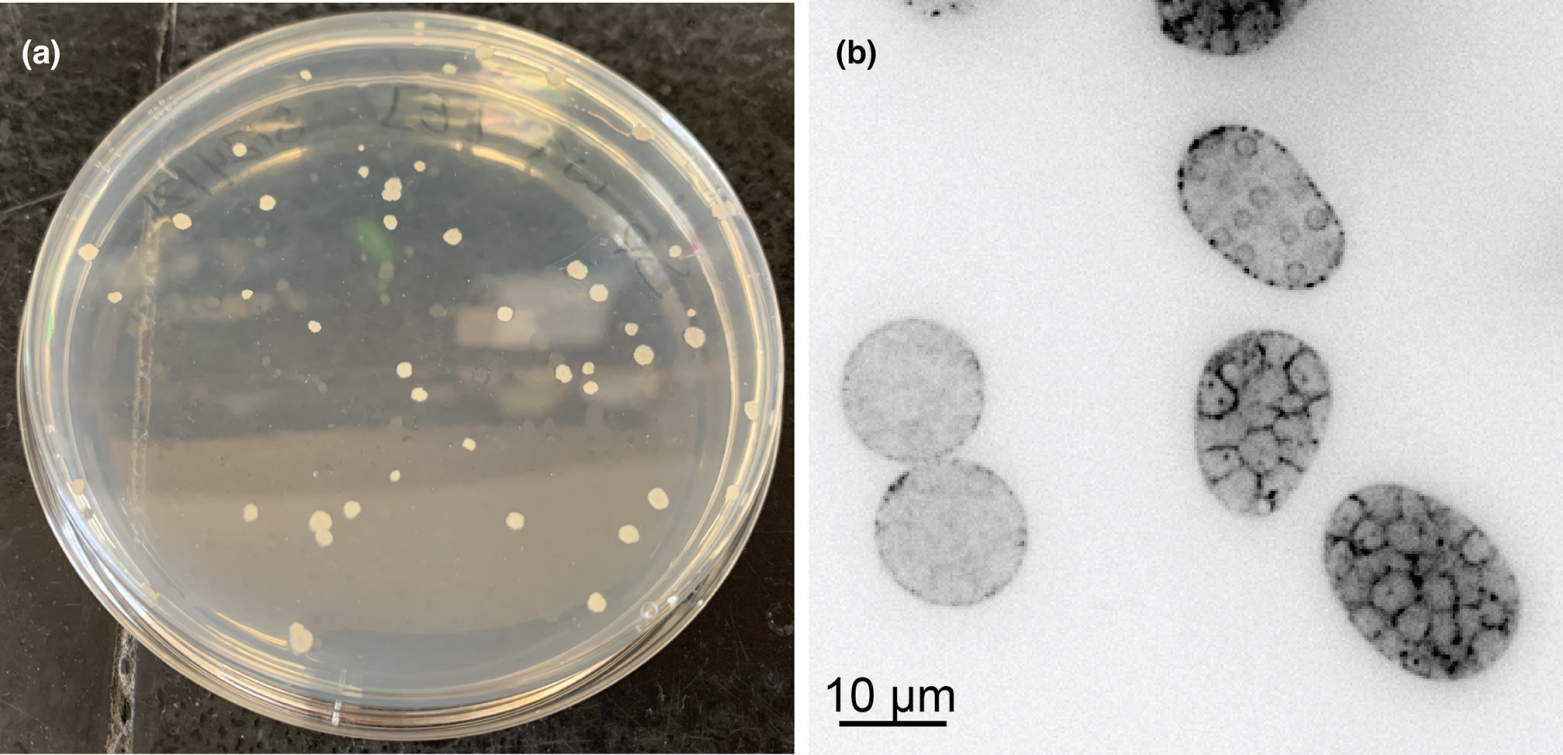
Colonies of transformed *Spizellomyces punctatus* and a Lifeact-tdTomato strain. (**a**) Colonies of transformed *Spizellomyces punctatus* (*S.p*.), grown on selective K1 media for 4 days at 28°C. Colonies are rough, opaque and off-white. (**b**) Fluorescent images of a strain of *S.p*. stably expressing Lifeact-tdTomato that highlights polymerized actin (black).

After harvesting the co-culture, transformed *S.p*. can be selected for. If transformation was successful, colonies of transformants should appear on selective K1 media after about 4 days at 28°C (Fig. 5a). These colonies should be rough, opaque, and off-white, and can number from a few to hundreds.

### Picking colonies of transformed *Spizellomyces punctatus*

Once a colony is picked and plated onto a new selective K1 plate, the resulting growth after about 4 days at 28°C should appear similar to the growth seen on the IM plates (Fig. 4b). When viewed under a microscope, zoospores should be swimming and there may be some larger-than-normal sporangia, this is a typical response to transformation for *S.p*. No bacteria should be present on the plates of isolated transformants.

Transformants should be verified through molecular analysis and microscopy. Here, we provide an example of *S.p*. transformed with LifeAct-tdTomato to confirm that the transformation was successful (Fig. 5b).

## Future directions

As the only genetically tractable chytrid species at the time of this publication, transformation of the free-living species *S.p*. is a vital part of the toolkit for studying chytrid cell biology, broadening the knowledge on an ecologically important group of fungi. The approach detailed here facilitates random chromosomal integration of a genetic cassette, which allows for overexpression of (fluorescent) fusion proteins and/or testing promoter activity. Random integration, however, does not lend itself easily to targeted gene disruption. For that approach, we are hopeful that the recent development of high-efficiency cargo delivery by electroporation may be used to develop targeted gene disruption [15].

Chytrid fungi are genetically diverse and include over a thousand species. Adapting transformation protocols to other chytrid lineages will enable new lines of research. For example, expanding the toolkit for genetic manipulation to the frog-killing chytrid *Batrachochytrium dendrobatids* and other parasites could reveal molecular mechanisms used by chytrids to parasitize their hosts. Moreover, we look forward to molecular genetic tools being developed for multiple chytrid lineages to facilitate testing hypotheses regarding the evolution of fungi, and answer other key questions about these fascinating and ecologically important organisms.

## Supplementary Data

Supplementary material 1
