# Supplementary material for: A guide to Agrobacterium-mediated transformation of the chytrid fungus Spizellomyces punctatus
Source: Access Microbiol. 2023 May 11;5(5):acmi000566.v3. doi: 10.1099/acmi.0.000566.v3 (PMC10267658; doi:10.1099/acmi.0.000566.v3)
Supplement: Supplementary material 1 [file acmi-5-566.v3-s001.pdf]

Collection of protocols title: *Agrobacterium*-mediated transformation of the chytrid fungus *Spizellomyces punctatus*

Authors on all protocols: Sarah M Prostak, Edgar Medina, Erik Kalinka, and Lillian Fritz-Laylin

Protocol 1: Electroporation of *Agrobacterium tumefaciens* with a plasmid of interest.

Abstract: Electroporation is a widespread method of transforming competent *Agrobacterium tumefaciens* (*Agro*) cells with a plasmid containing a T-DNA of interest. The resulting *Agro* can be used to transform various plants and fungi, resulting in transformed cell lines. This protocol outlines the standard electroporation protocol we use to transform *Agro* in preparation for *Agrobacterium*-mediated transformation of the chytrid fungus *Spizellomyces punctatus.*

Guidelines: Any binary plasmid that works in *Agrobacterium tumefaciens* strain EHA105 (GoldBio #CC-225-5x50) can be used for this procedure. We have had success using plasmids derived from pPZP201-BK (PMID: 7919218). It is imperative that all steps be carried out at 4℃ up until electroporation. Ensure proper sterile technique throughout this protocol; perform all steps except centrifugation and electroporation charge delivery in a laminar flow hood or in the sterile area around an open flame.

Before starting: Electroporation should occur at least 4 days prior to the intended *Spizellomyces* transformation time to ensure that active, single colonies are available to be selected and transferred to liquid culture the night before transformation day (see Protocol 3).

<u>*Materials:*</u>

Use a fresh streak of *Agrobacterium tumefaciens* EHA105 (GoldBio #CC-225-5x50) to spread a lawn into a LB plate, (a new streak from a -80°C stock will take ~48 hours to display visible growth at 28°C , plan accordingly).
LB agar plates (1.5% w/v) with and without selection antibiotics, sterile (see recipe)
Purified plasmid(s) of interest resuspended in molecular biology grade water
Molecular biology grade water, sterile such as MilliQ or equivalent
SOC medium (Invitrogen #15544-034)
10% (v/v) Glycerol, sterile (Fisher #G33-500)
1.5 mL centrifuge tubes, sterile (such as Fisher #02-681-331)
0.5 mL centrifuge tubes, sterile (such as Fisher #02-681-268)
2 mm electroporation cuvettes, sterile (Bulldog #12358-346)
Culture tubes, sterile (such as VWR #60818-703)
100-1,000 µL micropipette such as Eppendorf #3123000063
20-200 µL micropipette such as Eppendorf #3123000039
0.1-2.5 µL micropipette such as Eppendorf #3123000012
Filter tips for the micropipettes, sterile such as USA Scientific #1122-1830
5 mm Glass beads, sterile
Ice bucket with ice
Centrifuge capable of cooling to 4℃
Laminar flow hood and/or open flame, for maintaining sterility.

70% (v/v) ethanol for maintaining sterility (if using laminar flow hood)
Exponential decay electroporator such as Gene Pulser Xcell (Bio-Rad #1652660)
Shaking incubator at 28℃

Steps

1. Cool the following materials on ice at least 20 minutes prior to starting:
    a. Plate with a lawn of wild-type *Agrobacterium* grown overnight at 28C.
    b. Purified plasmid(s) of interest
    c. Molecular biology grade water, sterile
    d. 10% (v/v) glycerol, sterile
    e. 1.5 mL centrifuge tube(s), enough to hold the volume of *Agro* harvested.
    f. 0.5 mL centrifuge tube(s), one per plasmid to be transformed, plus controls
    g. 2 mm electroporation cuvettes, one per plasmid to be transformed, plus controls
2. Add 1-2 mL of ice-cold water to the plate of *Agrobacterium*. Hold the plate at ~45 degrees and run the water over the surface at least 3 times, gently scraping along the agar if necessary to recover the lawn of bacteria. Try to not drag too many big clumps. The resulting harvest should have the consistency, color and density of whey.
3. Transfer the 1 mL of harvested cells to a 1.5 mL centrifuge tube, immediately place back on ice.
4. Pellet the cells at 4000 rcf for 5 minutes in a rotor prechilled to 4℃.
    If in a pinch, a rotor for a centrifuge without cooling capabilities can be stored overnight at 4℃ *or for 10 minutes at -20*℃.
5. Remove the supernatant and gently resuspend the cells in 1 mL of water. Do not vortex.
    Keep cells on ice when not in use.
6. Repeat steps 4 and 5, 2 more times for a total of 3 washes.
7. Remove water and resuspend the cells in 800 μL of cold 10% (v/v) glycerol, place tubes back on ice.
8. Add 50 μL of cells to new 0.5 mL tubes, place back on ice.
    Use one 0.5 mL tube for each plasmid to be transformed, plus more for planned controls.
9. Add 1 μL of the plasmid of interest (200-300 ng/uL; directly from a miniprep should work) to its appropriate tube of cells, place back on ice and mix gently by pipetting .
    Use 1 μL of water as a negative control.
10. Transfer the cells to cold 2 mm electroporation cuvettes, place back on ice.
    Again, use one cuvette for each control or plasmid to be transformed individually.
11. Prepare the recovery media in advance by placing 150 μL of room temperature SOC medium to one 15 mL culture tube for each electroporation.
12. Turn on the electroporator and create an electroporation program with the following settings:
    a. Voltage= 2400V
    b. Capacitance = 25 μF
    c. Resistance = 200 Ω
    d. Cuvette size = 2 mm

13. Fully dry the cuvette before placing it into the electroporator chamber.

    Failure to fully dry the cuvette will lead to current arcing and improper electroporation.

14. Electroporate your cuvette.

15. As quickly but as gently as possible, remove a little less than 150 µL of SOC medium from the appropriate tube for the plasmid and pipette it into the cuvette. Remove the full volume from the cuvette and return it to the original culture tube. Do this by a flame and with good sterile technique.

    The best way to do this is to set a p200 to 150 µL, but to not pull the entire volume, leaving enough space left to fit the 50 µL that is in the cuvette.

16. Incubate culture tubes at 28°C, shaking at 225 rpm for 4 hours

    *Meanwhile, place the appropriate number of LB plates with and without selection antibiotics to pre-warm at 28°*C

17. Add 4-6 sterile glass beads to each LB plate
18. Add 10 µL cells to the appropriate plates

    To make spreading this small volume easier, add 40 µL sterile water to the middle of the plate before adding the cells

    The electroporation efficiency for this protocol is very high: ≥1.6 x 10e8 cfu/µg pCAMBIA1391z DNA (GoldBio) or 1.25x10e5 cfu/µg plasmid (PMID: 29487777)

    Do not add more than 10 µL of cells or you risk overgrowth and a lack of individual colonies.

19. Seal and invert the plates and incubate them at 28°C for about 4 days

    Colonies should appear within 4 days. If colonies of appreciable size (2-3 mm) appear earlier than that, continue on with Sp transformation

    Grow any colonies of interest in liquid media and freeze 25% glycerol stocks to avoid needing to re-electroporate Agro.

Protocol 2: Culturing *Spizellomyces punctatus* (*Sp*) prior to transformation day.

Abstract: In order to increase transformation efficiency, a more synchronous culture of *Sp* is needed. This process involves subculturing active *Sp* plates in the days leading up to transformation day. At least two generations of *Sp* should be grown on antibiotic free K1 medium to ensure no trace antibiotics are present in the culture for transformation day. Proper sterile technique should be followed at all times when maintaining cultures of *Sp*; use either a laminar flow hood or work in the sterile area around an open flame.

Before starting: In order to prepare enough plates to provide an adequate amount of zoospores for transformation, subculture one plate of *Sp* per plasmid to be transformed. One active plate is enough to subculture 2-3 new plates. Complete this protocol roughly 36 hours and then again roughly 18 hours prior to the intended transformation time.

Materials

Active, healthy culture of wild-type *Spizellomyces punctatus* (ATCC 48900)
K1 agar plates (1.5% w/v) without any antimicrobial (see recipe)
DS solution (see recipe)
50 mL conicals, sterile (such as CellTreat #229421)
100-1,000 µL micropipette such as Eppendorf #3123000063
Filter tips for the micropipette, sterile such as USA Scientific #1122-1830
20 mL luer-lock syringes (such as Fisher #22-124-967)
25 mm syringe filter holders (Fisher #NC9972954), preloaded with Grade 1 Whatman paper (Fisher #09-927-223), sterile
Laminar flow hood, or open flame to maintain sterility
70% (v/v) ethanol for maintaining sterility (if using laminar flow hood)
Incubator at 28℃

Steps

1. Flood enough active *Sp* plates each with 1 mL of DS to fit your needs

   One active plate is enough to seed 2-3 new plates and you will need one plate of active Sp per plasmid to be transformed on transformation day.
2. Holding the plate at an angle, gently wash the 1 mL of DS over the agar at least 3 times.
3. Collect all zoospores into a 50 mL conical.
4. Filter all zoospores into a new 50 mL conical using the sterile 25 mm syringe filter preloaded with Whatman Grade 1 filter paper.
5. Inoculate the proper amount of new K1 plates for your needs with 250-500 µL of filtered zoospores.

   The amount of spores transferred to new plates depends on the density of the plate being harvested, be careful not to overcrowd the plates, as zoospore release will decrease.
6. Add 500 µL of fresh DS to the newly inoculated plates
7. Gently tilt the plates to spread the liquid across the entire surface of the agar.
8. Incubate at 30℃ in a humidity chamber

   Using a humidity chamber is standard practice, as it prevents the agar from drying out. Placing the plates into a plastic bin with a beaker of ~20 mL of water should be enough.

   Complete this protocol in its entirety 36 and 18 hours prior to intended transformation time.

Protocol 3: Growing liquid cultures of *Agrobacterium* prior to transformation day

Abstract: Overnight liquid cultures of *Agrobacterium* transformed with the plasmid(s) of interest must be prepared the night before *Spizellomyces* transformation day. Liquid cultures must be inoculated from active plates/colonie. If you would like to use 25% glycerol stocks of previously transformed *Agrobacterium*, streak the desired cells onto the appropriate LB+selection plate, let it grow for 2-4 days at 28℃, then use this plate to inoculate the liquid media at least 12 hours before the intended transformation time. Ensure proper sterile technique throughout the protocol, using either a laminar flow hood or working in the sterile area around an open flame

Before start: Perform this protocol the night before your intended *Spizellomyces* transformation time.

<u>Materials</u>

Active plate of wild-type *Agrobacterium tumefaciens* EHA105 (GoldBio #CC-225-5x50), grown at 28℃ for at least 48 hours.
Active plate(s) of *Agrobacterium tumefaciens* EHA105 (GoldBio #CC-225-5x50) transformed with your plasmid(s) of interest (see Protocol 1), grown at 28℃ for at least 48 hours.
LB liquid media with and without selection antibiotics, sterile (see recipe)
Culture tubes, sterile (such as VWR #60818-703)
Inoculation loops (such as Fisher #22-363-602; pipette tips are also an acceptable alternative)
Laminar flow hood, or open flame to maintain sterility
70% (v/v) ethanol for maintaining sterility (if using laminar flow hood)
Shaking incubator at 28℃

Steps

1. Fill the appropriate amount of culture tubes each with 5 mL of LB liquid media

   Use one culture tube per plasmid or control. Double check you are using the proper selection antibiotic (or lack thereof) for the Agro strain to be grown.
2. Using an inoculation loop or pipette tip, gently scrape the surface of the appropriate *Agro* plate to collect the bacteria or pick the colony of interest
3. Swirl the loop or tip to dislodge the bacteria into the media of the appropriate culture tube.
4. Incubate at 28℃, shaking at 225 rpm for 12 hours, or overnight.

Protocol 4: Creating depressions in induction media plates

Abstract: Transformation is carried out by co-culturing *Agro* and *Spizellomyces* on induction media (“IM”). Once its virulence genes are induced, *Agro* will infect *Spizellomyces* and the plasmid the transfer DNA is randomly incorporated into the *Spizellomyces* genome. The IM media and agar plates (see recipes) should be no more than 1 month old, as the virulence inducer Acetosyringone is likely to degrade, decreasing its effectiveness. For each plasmid, four ratios of *Agro* and *Sp* will be made and plated onto one plate (See Protocol 5.3). Prepare IM plates with small depressions using a glass culture tube to keep the co-culture liquids from touching each other.

Before start: The protocol below outlines how to prepare the depressions and should be practiced until the agar is no longer scarred. Prepare these plates within a month of your intended *Spizellomyces* transformation time.

Safety warnings: Protective eye goggles must be worn during this procedure, as the potential for broken or cracked glass is a hazard.

<u>Materials</u>
Induction Media agar plates (1.5% agar w/v; see recipe)
Large diameter glass culture tube (>10mm, such as Fisher #14-957N)
50 mL conical (such as CellTreat #229421)
70% ethanol to maintain sterility
Open flame source to maintain sterility

Steps

1. On the bottom, divide an IM plate into quadrants
2. Draw circles 2.5 mm in diameter in each quadrant
   Ensure the circles do not overlap
3. Place a 20-30 mm diameter glass culture tube into a 50 mL conical tube
4. Add enough 70% ethanol to cover the first inch of the culture tube
5. Burn off the ethanol on the tube using a flame, let the glass cool in the sterile area around the open flame
6. Dip the culture tube back into the ethanol
7. Repeat steps 3-5 a total of 3 times
8. Working within the sterile area around the flame, gently press the warm (but not enough to melt the agar), sterile culture tube into the agar at the center of one circle drawn earlier
9. Gently press on the agar and move the culture tube in circles until a depression is formed roughly the size of the circle drawn
   Ensure the four circles of one plate do not overlap and can hold about 200 µL of liquid.
   Depressions can be seen by holding the plate to light at an angle; the light should bend around the circle.
10. Repeat for all quadrants of all IM plates needed.

Protocol 5: *Agrobacterium*-mediated transformation of *Spizellomyces punctatus*

Abstract: Once you have completed protocols 1-4, you are ready for transformation. This protocol is lengthy, requires accurate timing, and takes a minimum of 4 days to see results. Zoospores will need to be harvested from the plates prepared in Protocol 2; the process of harvesting is described below. Once *Agro* is at the appropriate OD, transformation is carried out by co-culturing *Agro* and *Sp* on induction media ("IM") to induce the expression of *Agro's* virulence genes and transformation of *Sp* cells. Co-culturing must be done on IM plates with premade depressions in the agar (see Protocol 4).

Guidelines: This section outlines the transformation process from growing *Agro* to the end of co-culturing. For harvesting the co-culture and selecting transformants, see Protocols 6 and 7. All steps of this protocol, except necessary centrifugation, must be carried out in a sterile environment, either in a laminar flow hood or in the sterile area around an open flame.

Before start: On transformation day, *Agro* must be diluted to an OD660 of 0.15 and then grown to an OD660 of 0.6. Under our laboratory conditions, this takes about 4-6 hours, but this time should be empirically tested for each lab before starting to ensure proper and repeatable results. Be sure to take this growth time into account when planning all steps on and prior to transformation day.

<u>Materials</u>

Overnight liquid cultures of desired *Agrobacterium tumefaciens* strains (see Protocol 3 for transforming *Agro*)
Active plates of wild-type *Spizellomyces punctatus*, grown without antibiotics (see Protocol 2)
IM liquid (see recipe)
IM plates with depressions (see Protocol 4)
DS solution (see recipe)
Culture tubes, sterile (such as VWR #60818-703)
50 mL conical tubes, sterile (such as CellTreat #229421)
1.5 mL centrifuge tubes, sterile (such as Fisher #02-681-331)
Pipette aid
Serological pipettes
100-1,000 µL micropipette such as Eppendorf #3123000063
20-200 µL micropipette such as Eppendorf #3123000039
Filter tips for the micropipettes, sterile such as USA Scientific #1122-1830
M4 repeat pipettor, such as Eppendorf #4982000322
Combitips for the repeat pipettor, sterile such as Eppendorf #0030089804 or similar
40 µm mesh filters (Fisher #22-363-547)
25 mm syringe filter holders (Fisher #NC9972954), preloaded with Grade 1 Whatman paper (Fisher #09-927-223), sterile
Plastic storage containers, or equivalent (e.x. plastic shoe box)
Centrifuge capable of holding 50 mL conicals (such as Eppendorf 5810R)
Laminar flow hood and/or open flame, for maintaining sterility.
70% (v/v) ethanol for maintaining sterility (if using laminar flow hood)
Shaking incubator at 28℃

Steps– 5.1 Growing Agro to the appropriate OD660

1. Dilute each overnight *Agro* culture to an OD660 = 0.15 using IM

In our experience, this works out to be 500 µL of overnight culture that has been spun for 5 min at 4500 rcf and then resuspended into 4 mL of IM. Dilution conditions should be empirically tested for each laboratory.

2. Grow the *Agro* at 28℃ and shaking at 225 rpm until OD660 = 0.6

   Under our laboratory conditions, this takes about 4-6 hours. Growth time to reach optimal OD660 should be empirically tested for each laboratory.

Steps– 5.2 Harvesting *Sp* zoospores for transformation

1. Flood all wild type *Sp* plates with 1mL of DS or IM for 1 hour.

   Do this about one hour before Agro has reached an OD660 of 0.6

   The solution used should have little to no effect on transformation success.
2. Harvest *Sp* zoospores by running 1 mL of fresh DS or IM along the agar of one plate, holding the plate at a 45 degree angle.

   Do this twenty minutes before Agro has reached an OD660 of 0.6
3. Take the liquid from the first plate and use it to harvest the zoospores on a second plate in the same manner.
4. Do this for all plates of *Sp* that were flooded in step 5.2.1

   If the volume becomes too much to hold in the serological pipette, empty the contents into a 50 mL conical tube (or other appropriately sized tube), then use 1 mL of this zoospore suspension to continue harvesting the remaining plates.
5. Pool all zoospores into a 50 mL conical (or other appropriately sized tube).
6. Pass the zoospore suspension through a 40 µm mesh filter into a new 50 mL conical tube.

   This removes clumps of cells that could clog a syringe filter.
7. Use a 18 gauge needle and an appropriately sized syringe to take up all of the 40 µm-filtered zoospore suspension.

   Be very gentle, the needle may damage the cells.

   Alternatively: Directly load a 25 mL syringe with the 40 µm-filtered zoospore suspension by using a p1000 set to 1000 µL and gently pushing the cells into the syringe through the tip.

8. Use a sterile 25 mm syringe filter preloaded with grade 1 Whatman paper to further filter the spores into a new 50 mL conical tube (or other appropriately sized tube).

   PRESS SLOWLY, or risk damaging spores. As a rule of thumb, do not filter more than 5 mL of zoospores for one syringe filter.

   This suspension is now to be used in co-culturing and should have a concentration of zoospores between 1-10 x10^6 cells/mL

Steps– 5.3 Co-culturing *Agro* and *Sp* in varying ratios

1. Per each control and plasmid to be transformed, prepare and label four microcentrifuge tubes according to Figure 3.
2. Pipette the appropriate amounts of IM and the final solutions from protocols 5.1 and 5.2 into the appropriate microcentrifuge tube in the order they appear in Figure 3.

   This will prevent cross contamination between strains

   *Pipetting time can be sped up by using an M4 repeat pipettor (*Eppendorf #4982000322*) to dispense 50 µL volumes at a time, ensuring the proper amount is dispensed and that the liquid does not splash due to the force of dispensing. This is best done by dispensing along the tube walls just above the water line.*

3. Gently pipette to mix the contents of the microfuge tube.
4. Transfer all 200 µL of an *Agro-Sp* co-culture to one of the premade depressions on a room temperature IM plate.

   All 4 co-culture tubes for one plasmid can be placed onto one IM plate. One tube per quadrant. Start on the left side and then go to the right side to lower the risk of contamination (or the opposite for left handed people).

5. GENTLY slide the IM plates to a place where they will not be disturbed

   Avoid picking up the plates, as this may result in the pools of co-culture to combine, disturbing the ratios established and potentially lowering transformation efficiency or success.

6. Leave the plates to dry at room temperature for 12-24 hours.
7. Seal plates with parafilm, invert, and grow in a closed chamber (such as a plastic storage container) at room temperature for 4 days.

   Evidence of growth appears as an opaque, roughly circular area on the agar. If not all of the quadrants have obvious growth, that is okay. As long as there is at least one quadrant that shows growth, Protocol 6 can be done, though success at gaining selectable transformants may be lower.

Protocol 6: Selecting for *Spizellomyces punctatus* transformants

Abstract: Once the co-culture IM plates have grown for four days, it is time to select Sp transformants. Selecting these transformants requires harvesting the cells from the co-culture plates, dislodging and removing any remaining *Agro* from the culture, and plating *Sp* onto K1 selection media. In our laboratory, we use 300 mg/L Hygromycin B (Gibco #10687010) as our selection marker. Additionally, we add 50 mg/L Carbenicillin and 50 mg/l Tetracycline to our K1 plates to kill *Agrobacterium*. All steps here, with the exception of centrifugation steps, must be carried out in a sterile environment, ideally in the sterile area around an open flame.

Safety warning: This protocol involves the use of single-edge razor blades. It is extremely important that proper safety protocols regarding sharps be taken– protective eye equipment and a sharps container must be used while conducting this protocol and disposing of razors.

Materials

Co-culture of *Agro* and *Sp* after 4 days of growth (see Protocol 5)
Single edge razor blades, sterile (such as Personna .009 RD #94-120-71)
K1 plates with and without selection antimicrobials
Forceps (such as AESCULAP BD232)
DS solution, sterile (see recipe)
50 mL conical tubes, sterile (such as CellTreat #229421)
100-1,000 µL micropipette such as Eppendorf #3123000063
20-200 µL micropipette such as Eppendorf #3123000039
Filter tips for the micropipettes, sterile such as USA Scientific #1122-1830
Vortexer such as Fisher #14-955-163
Centrifuge capable of holding 50 mL conicals (such Eppendorf 5810R)
Plastic storage containers, or equivalent (e.x. plastic shoe containers)
Open flame source

Steps

1. For each plasmid transformed, prepare a 50 mL conical with 30 mL of DS solution.
2. From the appropriate conical for the plate to be harvested, remove 1 mL of DS.
3. Distribute the DS among the four quadrants of the corresponding IM plate.
4. Incubate at room temperature for 5 minutes.
5. Using forceps, sterilize both sides of a single edge razor blade with the flame.
6. Tilt the rehydrated IM plate and gently scrape the surface of the agar and collect the growth into the DS pooled at the bottom.
7. Rotate the plate and scrape along the agar until most of the opaque areas on the plate are gone.
8. Use a 1000 µL pipette tip to scrap any remaining growth from the razor blade and resuspend the growth to the appropriate conical tube filled with DS.

   Once the growth is taken off the razor blade, the blade should be thoroughly sterilized using the flame and then discarded into a sharps container.
9. Aspirate all of the liquid from the scraped plate and slowly dispense the liquid back into the appropriate conical tube.

   A density gradient between the heavier cell suspension and the lighter DS solution will form. Maintaining this gradient at this pointby placing retrieved co-culture in the bottom of the tube. This can help you continuously retrieve "clean" DS to continue harvesting the plate more easily.
10. From the top of the density gradient, remove 1 mL of DS.
11. Wash the surface of the scraped IM plate several times with this fresh DS.
12. Return the volume back to the conical.
13. Invert the conical 3 times.

14. Vortex the conical for 1-2 seconds to dislodge any remaining *Agro* from *Sp* cells.
15. Repeat steps 2-14 for each plasmid transformed (i.e, for each IM plate you have)
16. Centrifuge the conical(s) for 10 minutes at 2000 rcf at room temperature.
17. Check for the presence of a pinkish pellet, this is the chytrid *Sp*.
18. Gently pour off the supernatant with the high side of the pellet facing up.
    This step can result in a great loss of Sp cells, thus pouring with this orientation will reduce the amount of cells lost.
19. Gently resuspend the pellet into 500 µL of fresh DS.
20. Pipette 200 µL of the resuspended *Sp* onto a K1 plate with selection antibiotics.
    We use 300 mg/L Hygromycin B (Gibco #10687010) and 50 mg/L Carbenicillin and 50 mg/L Tetracycline to prevent further *Agro* growth
21. Spread the cells using 4-5 sterile glass beads.
22. Once the plate is dry, remove the glass beads.
23. Seal and incubate plates in a humidity chamber at room temperature for 4 days or until colonies appear on the plate.

Protocol 7: Picking colonies of transformed *Spizellomyces punctatus* (*Sp*)

Abstract: If transformation was successful, antibiotic-resistant Spizellomyces colonies should appear after 4-6 days of growth on selection media. These colonies should be rough, white, and opaque. When viewed under a microscope, there should be clustering of sporangia and some movement of zoospores. Picking these colonies and culturing them until there are enough cells for cryopreservation and to use in downstream molecular analysis is the last protocol in the entire transformation process. All steps here, with the exception of centrifugation steps, must be carried out in a sterile environment, either in a laminar flow hood or in the sterile area around an open flame.

Safety warning: proper handling of 18G needles must be observed to avoid personal injury. All sharps should be disposed of into a proper sharps container.

<u>Materials</u>

Active plate of colonies of *Sp* transformants (see Protocol 6)
DS Solution, sterile (see recipe)
18G needle, sterile (BD #305196)
1.5 mL microcentrifuge tubes, sterile such as Fisher #02-681-331
K1 agar plates (1.5% w/v) with selection antimicrobials, sterile (see recipe)
20-200 µL micropipette such as Eppendorf #3123000039
Filter tips for the micropipettes, sterile such as USA Scientific #1122-1830
Laminar flow hood and/or open flame, for maintaining sterility
70% (v/v) ethanol for maintaining sterility (if using laminar flow hood)

Steps

1. Divide one K1 plate with selection antimicrobials into four sections using a marker on the bottom of the plate.

   One plate can be used to plate up to 4 colonies for one plasmid transformation. This reduces materials used in the initial amplification stage.
2. Aliquot 50 µL of DS into one microcentrifuge tube per colony to be picked.
3. Using an 18G needle, gently lift the colony of interest from the agar.

   Be careful not to poke too deeply into the agar. The goal is to lift as little agar as possible from the plate, while picking up most of the colony.

   To prevent cross contamination between colonies, either flame the needle between picking colonies, or use a new, sterile needle for each colony.
4. Resuspend the colony into the appropriate tube pre-filled with DS.

   Swirl the needle gently, but with enough force to dislodge the colony from the needle
5. Pipette gently to mix and break up the pellet.
6. Pipette 50 µL of the resuspended colony onto one quadrant of the K1 plate prepared in step 1.
7. Repeat steps 3-6 for each colony to be picked.
8. Let the cells grow for 2-3 days before rehydrating the quadrants with 100 µL of DS and transferring each colony to its own quadrant of a new K1 selection plate.

   Only a small portion of the quadrant is needed for colony expansion.
9. After 2-4 rounds of colony expansion there is enough inoculum to transfer each isolated colony into its own plate.
10. Continuing subculturing each colony until enough culture exists to freeze and perform downstream analyses.

Reagents and Solutions: *Agrobacterium*-mediated transformation of *Spizellomyces punctatus*

Section 1: Dilute Salts Stock Solution I (10x)

0.5 mM KH_2PO_4 (68.05 g) (Potassium Phosphate monobasic; Sigma #P0662)

0.5 mM K_2HPO_4 (87.09 g) (Potassium Phosphate dibasic; Sigma #P3786)

0.5 mM $(NH4)_2HPO_4$ (66.04 g) (Ammonium Phosphate dibasic; Sigma #215996)

500 mL Water

Sterilize by filtration

Store at room temperature for up to 12 months

Section 2: Dilute Salts Stock Solution II (10x)

0.05mM $MgCl_2$ (25.42 g) (Magnesium chloride; Sigma #M8266)

0.05mM $CaCl_2$ (18.38 g) (Calcium chloride; Sigma #C1016)

250 mL Water

Sterilize by filtration

Store at room temperature for up to 12 months

Section 3: Dilute Salts Solution (1x) (Machlis, 1958)

500 µL DS Stock Solution I

100 µL DS Stock Solution II

1 L sterile water

Prepare solution in a sterile laminar flow hood with sterile supplies

Store at room temperature for up to 12 months

Section 4: K1 Media (liquid and solid)

0.06% Bacto Peptone (0.6 g) (w/v; BD #211677)

0.04% Yeast Extracts (0.4 g) (w/v; Fisher #BP1422-2)

0.18% Glucose (1.8 g) (w/v; Sigma #G5767-5KG)

For solid media only: 1.5% (w/v) agar (15 g) (Fisher #BP1423-500)

Water up to 1 L

Sterilize by autoclaving

Let cool to 60°C before adding any selection antimicrobials

Store at 4°C for up to 6 months

Section 5: LB media (liquid and solid)-- made from individual components

1% Tryptone (10g) (w/v, Sigma #T7293)

1% NaCl (10g) (w/v; Sodium Chloride; Fisher #BP358-1)

0.5% Yeast Extract (5 g) (w/v; Fisher #BP1422-2)

For solid media only: 1.5% (w/v) agar (15 g) (Fisher #BP1423-500)

Water up to 1 L

Sterilize by autoclaving

Let cool to 60°C before adding any selection antimicrobials

Store at 4°C for up to 6 months

***Section 6:* LB media (liquid and solid)-- commercially available**

25 g LB powder (Sigma #L3522-1KG)

For solid media only: 1.5% agar (15 g) (w/v; Fisher #BP1423-500)

Water up to 1 L

Sterilize by autoclaving

Let cool to 60°C before adding any selection antimicrobials

Store at 4°C for up to 6 months

Section 7: Minimal Salts Solution (2.5x)

26.6 mM KH_2PO_4 (3.625 g) (Potassium Phosphate monobasic; Sigma #P0662)

29.4 mM K_2HPO_4 (5.125 g) (Potassium Phosphate dibasic; Sigma #P3786)

6.4 mM NaCl (0.375 g) (Sodium Chloride; Fisher #BP358-1

5 mM $MgSO_4 \cdot 7H_2O$ (1.250 g) (Magnesium sulfate heptahydrate; Sigma #2303915)

1.1 mM $CaCl_2 \cdot 2H_2O$ (0.165 g) (Calcium chloride dihydrate; Sigma #C7902-500G)

22.3 µM $FeSO_4 \cdot 7H_2O$ (6.2 mg) (Iron(III) sulfate heptahydrate; Sigma #F8263-500G)

9.5 mM $(NH_4)_2SO_4$ (1.250 g) (Ammonium sulfate; Sigma #A2939-500G)

Water up to 1 L

No need to sterilize, precipitate is normal

Store at room temperature for up to 1 year

Section 8: MES with acetosyringone

40 M MES pH 5.3 (7.7 g) (2-(N-morpholino)ethanesulfonic acid; Sigma #M2933-500G)

200 µM acetosyringone (0.0392 g) (Sigma #D134406-5G)

pH with KOH

MES must be at pH 5.3 before adding acetosyringone

Water up to 50 mL after pHing

Filter sterilize, DO NOT autoclave

Add to IM recipe after other components are autoclaved and cooled

Section 9: Induction Media (liquid and solid)

1x Minimal salts solution (400 mL of 2.5x stock solution, see recipe above)

10 mM glucose (0.9 g) (w/v; Sigma #G5767-5KG)

0.5% glycerol (v/v; 5mL) (Fisher #G33-500)

For solid media only: 1.5% (w/v) agar (15 g) (Fisher #BP1423-500)

Water up to 950 mL

Sterilize by autoclaving BEFORE adding MES with acetosyringone

50 mL MES with acetosyringone (see recipe above; only add after autoclaving other components and cooling to 58℃)

DO NOT autoclave acetosyringone, this will degrade the hormone

Store at 4℃ for up to 1 month
